# Patient experience feedback in UK hospitals: What types are available and what are their potential roles in quality improvement (QI)?

**DOI:** 10.1111/hex.12885

**Published:** 2019-04-23

**Authors:** Claire Marsh, Rosemary Peacock, Laura Sheard, Lesley Hughes, Rebecca Lawton

**Affiliations:** ^1^ Bradford Institute for Health Research Bradford UK; ^2^ University of Leeds Leeds UK; ^3^ Present address: University of Leeds Leeds UK; ^4^ Present address: Health & Community Services Jersey UK

**Keywords:** accountability, consensus exercise, feedback, Friends & Family Test, hospitals, NHS Inpatient Survey, patient experience, quality improvement, wards

## Abstract

**Background & objectives:**

The comparative uses of different types of patient experience (PE) feedback as data within quality improvement (QI) are poorly understood. This paper reviews what types are currently available and categorizes them by their characteristics in order to better understand their roles in QI.

**Methods:**

A scoping review of types of feedback currently available to hospital staff in the UK was undertaken. This comprised academic database searches for “measures of PE outcomes” (2000‐2016), and grey literature and websites for all types of “PE feedback” potentially available (2005‐2016). Through an iterative consensus process, we developed a list of characteristics and used this to present categories of similar types.

**Main results:**

The scoping review returned 37 feedback types. A list of 12 characteristics was developed and applied, enabling identification of 4 categories that help understand potential use within QI—(1) Hospital‐initiated (validated) quantitative surveys*:* for example the NHS Adult Inpatient Survey; (2) Patient‐initiated qualitative feedback: for example complaints or twitter comments; (3) Hospital‐initiated qualitative feedback: for example Experience Based Co‐Design; (4) Other: for example Friends & Family Test. Of those routinely collected, few elicit “ready‐to‐use” data and those that do elicit data most suitable for measuring accountability, not for informing ward‐based improvement. Guidance does exist for linking collection of feedback to QI for some feedback types in Category 3 but these types  are not routinely used.

**Conclusion:**

If feedback is to be used more frequently within QI, more attention must be paid to obtaining and making available the most appropriate types.

## 1 INTRODUCTION

The importance of listening and responding to the voices of patients and carers as a means of supporting high quality and safe care in hospital settings has been strongly advocated.^1^, ^2^, ^3^ The use of patient experience (PE) feedback as a data tool within quality improvement (QI) is a seemingly logical step as evidenced by a systematic review^4^ into how different types of feedback have been used in QI, and a more discursive piece on PE feedback as measurement data.^5^ Both reveal an immature field of study providing more questions than answers, relating to what feedback to collect, and how and when, and then how to use feedback to inform and measure QI. This lack of certainty has not prevented its collection: we know that much feedback is collected but is not used.^6^ We also know that when staff are presented with feedback and encouraged to use it for QI, they are faced with a complexity of social and logistical barriers.^7^ In a period of shrinking resources when capability for data management and PE is notably stretched,^8^ we must improve our knowledge of the potential roles of different types of feedback in QI so that resources can be directed appropriately.

We note a number of specific uncertainties surrounding the potential for different types of PE feedback to be used in QI. Most apparent is the debate over the comparative value of quantitative and qualitative types. In the systematic review of uses of PE types,^4^ quantitative surveys were revealed to be the most frequently collected type of PE data (often mandated) but the least acceptable to health‐care teams with respect to use within QI, considered by many to reflect PE as conceived externally, rather than providing useful information for improvement. Conversely, teams like more qualitative types of feedback (ie, more in‐depth accounts of individuals’ experiences) that are perceived to more closely portray their patients’ concerns. However, reviews have also identified an apparent sense of nervousness amongst hospital teams surrounding the use of qualitative data as it is regarded as time‐consuming to collect^4^ and difficult to interpret without bias.^5^

Second is the critique directed at the data source currently most readily available in England—the mandated Friends and Family Test (FFT). Whilst proponents argue it offers timely, continuous and local‐level data ripe for use in QI at many levels,^9^ others^10^ suggest that problems of validity and representation make comparisons between time and space impossible, and the lack of qualitative detail with which to contextualize results, mean this tool is not fit for purpose. Third is the growing interest in utilizing types of data that are not collected specifically by an organization for improvement, but are available for use should organizations wish. So, there is interest in utilizing complaints as data,^11^, ^12^ as well as online reviews^13^ but our understanding of these sources is embryonic, and some argue that organizations will struggle to engage with such sources if they did not seek, sanction or solicit them.^14^ Finally, there is the arrival of frameworks that link feedback collection to a QI change process in which involvement of staff and patients is generally high and localized. These include methods such as Experience‐Based Co‐Design for which evidence of impact is growing, but less evidence of cost‐effectiveness of this resource‐intensive technique relative to other QI processes is available.^15^

One response to this growing appreciation of the different types of PE feedback potentially available has been to suggest that health‐care staff should mix different (and multiple) types of PE data to triangulate and obtain the most comprehensive information for improvement.^5^ However, this ambition is also debated with one critique focusing on the loss of meaning that can arise if rich, “untameable” qualitative data are “aggregated” and “triangulated” with quantifiable data in an attempt to arrive at more tractable issues.^16^ Instead, it is argued that “softer” less quantitative data serve a different purpose—disrupting assumptions rather than counting occurrences. The assertion that qualitative PE feedback provides additional (not just supporting) insights necessary for understanding aspects of PE not possible to elicit through quantitative surveys has been made by others.^17^, ^18^ These are the “relational” aspects so important to concepts of PE (eg, how were you treated?) as opposed to more transactional components (eg, was a service provided on time?) targeted by surveys. With this in mind, triangulation and aggregation concepts provide no neat solutions to handling different types.

Amidst these uncertainties then, there have already been some attempts to distinguish between feedback types according to potential purpose. In 2013, an evidence scan^19^ outlined a wide range of PE feedback types available, from quantitative surveys to qualitative patient stories, and characterized them by their ability to generalize (quantitative types) or describe (qualitative types). Subsequently, there have been two reviews of quantitative PE surveys available worldwide—one^20^ assesses them for utility arguing that their primary use is for “high‐stake purposes” such as benchmarking, hospital rankings and securing funding. The other^21^ concludes similarly and also summarizes why they are not suitable for informing local (eg, ward, clinical team, disease group) improvement initiatives: they do not provide locally attributable data and they lack nuance and detail. However, the role of surveys in local‐level improvement has not been discounted altogether: it has been proposed that some surveys, if designed and supported to allow local interpretation and timely processing, could be used to monitor changes within local improvement process over time, in addition to benchmarking functions.^22^, ^23^

Indeed, the distinction between different uses of data within improvement is not a new one and in 1997, “The 3 Faces of Performance Measurement” were outlined: data used for accountability (outcome measurements of interest to external parties, eg, funders and regulators), data for improvement process (detailed information to aid identification of problems, opportunities for change and monitoring of success) and data for research (generating universal knowledge).^24^ We wish to build on all of these existing distinctions in order to guide improved utilization of the plethora of different PE feedback types that can now exist so that health‐care staff can make more informed choices about what can be achieved by engaging with them as data in improvement. To do so, we document the following three‐stage process that used UK hospitals as a case study, with which to understand types of data arising from different types of PE feedback:

1. A scoping review of all types of PE feedback currently available to hospital staff in the UK that builds on previous reviews of surveys to include other feedback available.
2. Development of a list of characteristics that we believe to be important in understanding potential use within QI that consolidates what is already known combined with our own research experience of improving quality of care.
3. Use of these characteristics to define types of feedback identified in our scoping review into distinct categories that can begin to inform policymakers, researchers and those responsible for collecting and using PE feedback, of their potential comparative uses.

Whilst we use NHS hospitals in the UK as a case study, we anticipate our characteristics list and categories to be relevant to types of PE feedback that arise in different hospitals elsewhere, but also as categories that could be used as a starting point to consider feedback in any health and social care setting where staff are considering how feedback can be used to improve services.

## 2 METHODS

### 2.1 A scoping review of sources of PE feedback in the UK

Between Spring and Autumn 2016, we conducted a scoping review comprising academic databases, grey literature databases and websites, and supported this with our own knowledge from the field and that of our study steering group. We also hand‐searched citations contained within returned documents. We identified surveys from the existing reviews^18^, ^19^ and then conducted our own search of academic databases to update and focus on the UK only. We used grey literature and websites to identify other types of PE feedback that we knew, because of their non‐validated status, were not likely to be found in academic journals, but more likely to be discussed in “guidance” documents and commentaries. We adopted a scoping review method, and not a systematic review, because flexibility of search terms within grey literature was paramount to enable as wide a range of PE feedback to be returned. Comprehensiveness of sources available in the UK, whilst important, was secondary to our aim of developing a characterization system and categories that we anticipate could be applicable to other types as they emerge. We were informed by a five‐step framework for conducting scoping reviews 25 as shown in Table 1.

**Table 1:** Five steps of our scoping review

Identifying the research question	“What sources of PE feedback are currently available to hospital staff in the UK?”
Identifying relevant studies	Search of academic databases (*Medline; Cinahl Plus; Amed; Scopus; Web of Science; Psych INFO; ProQuest Hospital collection*) using terms: ‘patient experience’*’patient’’ outcome assessment (healthcare)”, measures*. Timeframe: 2000‐2016. Search of grey literature (*Google, Google Scholar, Grey Literature Database, Royal College of Nursing database, Care Quality Commission (CQC), Collaborations for Leadership in Applied Health Research and Care (CLAHRC), Health Foundation, HealthTalk.org, iWantGreatCare, Health Watch, Kings Fund, NHS England, NHS Institute for Innovation & Improvement, NHS Surveys, Mumsnet, Patients Like Me, Patient Experience Portal, Patient Experience Network, Care Opinion, Picker Institute, Scottish Government, World Health Organization*) using terms ‘patient experience feedback within the NHS’, ‘patient experience feedback of hospital care’, ‘NHS use of patient experience feedback of hospital healthcare’, ‘improving patient experience’, ‘patient experience toolkit’. These were subsequently adapted to suit different ways organizations use terms. Timeframe: 2005‐2016—narrower than for academic databases due to high volume of returns. NB Different terms were used for academic databases than those for grey literature because of the different content likely to be returned through each route.
Study selection	Inclusion criteria: any sources of feedback relating to patient experience of hospital care; patient or carer perspective; for use in UK acute hospital setting. Exclusion criteria: sources of feedback relating to patient experience of specific aspects of quality such as safety, clinical outcomes, person centred care, performance of individual clinicians or health‐care staff, treatment/condition specific experiences; not patient or carer perspective; not secondary care; those under 18 y; for use outside UK.
Charting the data	The search returned 37 different types of PE feedback for which we immediately created 3 broad categories that were informed by our general understanding of the way feedback varied. This enabled the results to be displayed in 4 separate tables to aid comparison: Appendices S1a: (17 surveys), 1b (12 patient‐initiated feedback) and 1c (7 hospital‐initiated qualitative feedback). We found that 2 types of feedback did not fit well in any and placed these in a 4th Table as Appendix S1d (other). This was deemed a reasonably objective task and was therefore performed by one researcher (RP) with two additional researchers confirming these categories.
Collating, summarizing and reporting the results	Our categorization exercise, which is detailed below, fulfils this stage.

### 2.2 Developing a list of “defining characteristics”

We established a consensus team to develop a list of *12 key descriptive characteristics* to help understand the role of different feedback types in QI. This list is provided in Table 2. The team comprised the PI (Professor Psychology of Healthcare), 4 health service researchers (1 psychologist, 2 social scientists and 1 sociologist), 2 design researchers (concerned with presentation and usability of patient feedback), 1 health‐care improvement specialist and 1 patient involvement facilitator. The list developed iteratively through the following stages:

- The PI first used evidence referred to above, combined with own knowledge of QI and PE to produce an initial list of nine characteristics and presented this to the consensus team.
- The consensus team then added a further four characteristics to make 13.
- One researcher (RP) attempted to use this list to characterize six of the types returned through the review finding that twelve of the characteristics worked effectively and only one did not so this was removed. This was “whether the feedback only related to specific patient groups” which was not possible to ascertain from descriptions of the types.
- This list of 12 was then presented to the study steering group (comprising 4 lead researchers, 6 staff and 6 patient representatives from 3 hospital trusts, 2 national PE advisors) to ensure it made sense beyond the consensus team. This process led to clarification of the definitions and potential variability (character options) of each characteristic as listed in Table 2.

**Table 2:** Characteristics of PE feedback of relevance to QI

Characteristics of PE feedback	Character options
Nature of data obtained from feedback	
Type	Qualitative Quantitative Quantitative + comments Qualitative + star ratings
Level of applicability	Hospital Service or specialty Either
Evidence for validity (applies to surveys only)	Yes No
Timing of feedback	Whilst patient in situ Post‐discharge Either
Mode of feedback collection	
Mode of feedback collection	Survey (paper, telephone, Internet or combination) Internal hospital forms (web, paper) External (web, paper) Qualitative research methods (interviews, observation, focus groups)
Availability of feedback	
Requirement for feedback	Mandated by law or NHS Voluntary
Supporting hospital systems	Yes, formal system in place No formal system in place
Timeliness of feedback availability to service	Delayed “Real time”
Regularity of feedback	Continuous Annual or bi‐annual Ad hoc
Perspective captured	
Who initiatives feedback?	Patients or carers Service Provider (any level)
Who provides feedback?	Patient Carer Either
Defined role in QI	
Extent of the defined role	Data (potential data source only in words or numbers) Data + QI (has accompanying guidance on use within QI)

### 2.3 Assigning “characteristics”

These characteristics were then applied by RP to all returned types of PE, which enabled a definition of each type to be summarized into four broad categories within our Appendix S1 tables and described below. These were checked by two other members of the team before finalizing.

## 3 FINDINGS

We used our characteristics list to further understand and subdivide the feedback types within our initial four broad categories. This process also enabled us to provide more indicative titles for the categories than those we used as Appendix S1 titles. The categories and subcategories are shown in Table 3a‐d. The distinctions that we make between them are now described, highlighting potential implications for role within improving PE.

**Table 3:** (A) Category 1: Hospital‐initiated (validated)—Quantitative surveys; (B) Category 2: Patient‐initiated—Qualitative feedback; (C) Category 3: Hospital‐initiated—Qualitative feedback (D) Category 4: Other

(A)	
(a) Mandated	(b)Voluntary
*Hospital‐level:* The NHS Adult Inpatient Survey (England)^26^, ^27^, ^28^ Scottish Inpatient Patient Experience Survey^29^ Inpatient Patient Experience Survey (NI)^30^ *Any level:* Your NHS Patient Experience Survey (Wales)^31^ *Service or speciality:* NHS A&E Survey (England)^28^, ^32^ NHS Maternity Services Survey (England)^28^ Scottish Maternity Care Survey^33^	*Hospital‐level:* Hospital Care & Discharge^34^ *Any level:* PPE 15^35^ OxPIE^36^ Newcastle Satisfaction with Nursing Scale^37^ VOICE^38^ *Service or speciality:* PEECH 39 ICE Questionnaire^40^ New Models Study^41^ Urgent Care System^42^ Patient carer diary^43^

(B)	
(a) Formal hospital system	(b) No hospital system
Complaints^44^, ^45^, ^46^, ^47^ Liaison Service concerns^46^, ^47^, ^48^, ^49^, ^50^ Hospital‐supported feedback cards *Hospital‐supported websites*: NHS Choices^51^ iWantGreatCare^52^ Facebook set up by ward/hospital Care Opinion (if adopted)^54^	Compliments and Thank you cards^17^ *Websites:* Mumsnet^53^ Twitter Google reviews of hospitals Facebook (generally) Care Opinion (if not adopted)^54^ Other websites

(C)	
(a) Guidance linking data to QI process	(b) Focus on data presentation
Patient Journey^55^ Kinda Magic^56^ Experience‐Based Co‐design (EBCD)/Accelerated Experience‐Based Co‐design (AEBCD)^57^ Fifteen Steps challenge^58^ Always events^59^	Patient Stories^60^

(D)	
(a) Mandatory (England)	(b) Voluntary
Friends & Family Test (FFT)^61^	HowRWe^62^

### 3.1 Four categories of types of PE feedback

Seventeen types of feedback fitted into the first category “Hospital‐initiated quantitative surveys”.^26^, ^27^, ^28^, ^29^, ^30^, ^31^, ^32^, ^33^, ^34^, ^35^, ^36^, ^37^, ^38^, ^39^, ^40^, ^41^, ^42^, ^43^ Common to almost all of these is that data are predominantly quantitative, initiated by hospitals, targeting patients and not carers, with a significant delay (due to processing) in providing information back to the organization. However, closer inspection reveals a distinction between those that are mandated for high‐level organization use (either whole organization or for whole A&E or whole maternity departments) at regular but infrequent intervals, and those that are offered as voluntary tools for use as and when an organization decides. The former most clearly exhibit accountability features—providing organizational‐level data, within parameters defined and initiated by the organization, validated to make generalizations, comparisons (between organizations, or over time) when conducted for large samples. They are long—having over 70 items—and so require significant processing. On the other hand, with the exception of one (Hospital Care & Discharge^34^), the voluntary surveys can be applied at any level, at a timing to suit, or are especially designed for use within a local service or speciality (eg, ICE 40), without prescribing regularity. Unlike the mandatory surveys, only some are clearly validated. Many of these are significantly shorter—around 20 items. Potentially, these more flexible surveys that elicit local‐level information offer more scope for informing, or monitoring local improvement of PE. Only one survey (Your NHS Patient Survey Wales^31^) does not conform neatly to this subdivision. This survey is strongly recommended for use, not mandated, and is designed for use at any level. This implies more flexibility, and that perhaps it has been designed to inform or monitor local improvements, as well as to provide accountability.

We call the second category “Patient‐initiated qualitative feedback” and include 12 feedback types here^44^, ^45^, ^46^, ^47^, ^48^, ^49^, ^50^, ^51^, ^52^, ^53^, ^54^ that exhibit common traits: they provide qualitative data, applicable to any level of the organization, are initiated by patients on an ad hoc basis (whenever they choose to) and the feedback is available to the organization quickly (referred to as in real‐time). The concept of validity is not applicable because all data are provided on a case‐by‐case basis. Within this category, the significant distinction is between those types that are formally supported and those that are not. For those that are, this could be because they are mandated to do so (complaints^44^, ^45^, ^46^, ^47^; concerns^46^, ^47^, ^48^, ^49^, ^50^; NHS choices^51^), or because they choose to adopt a system (set up a ward‐based Facebook page or buy into iWantGreatCare^52^) to organize their feedback. Other types have no supporting system in place and include informal feedback (compliments, Thank you cards) that is received but not perceived of as data requiring attention or processing. We include a caveat here because some hospitals could have more formal systems for handling these (we know anecdotally that this happens) but this is not widely acknowledged or articulated as a process. This subcategory also includes websites external to the organization (eg, Facebook, Twitter, Mumsnet,^53^ Google reviews) where patients/carers may upload feedback, but there is no guarantee this will be viewed by hospital staff. Other less well‐known sites could also exist on the Internet. Care opinion^54^ currently spans both subcategories: it is offered as a formal system of data management for a fee if hospitals choose to adopt this. If not formally adopted, the platform can still be used by patients to upload feedback that may or may not be viewed by the hospital.

In summary, this category offers a different kind of “data” than that offered in Category 1, and therefore has a potentially different role within QI. In Category 1, feedback offers evidence‐based scope for use in benchmarking and monitoring of organizational trends. Category 2 feedback provides more local‐level information that would not be valid for use in that way. It exhibits some characteristics (nuance, specificity) that suggest potential use within local QI processes especially problem identification. Currently, however, feedback within this category is presented largely on a case‐by‐case basis and *not as collated data* ready to use. This makes its proposed role as a data source more tentative than the surveys of Category 1, and we return to this issue in the Discussion.

We name the third category “Hospital‐initiated qualitative feedback,” and this includes six types of feedback^55^, ^56^, ^57^, ^58^, ^59^, ^60^ with some common, defining features: feedback is predominantly qualitative and can be collected for any level of service by a variety of methods with varying degree of prescription in this regard. Interviews are common but focus groups, observation and shadowing all feature here. All types elicit rich data that takes time to process. All have a defined role within QI, albeit to a varied extent. Feedback collection is initiated by staff but in striking contrast to surveys (which cover issues deemed important to organizations about their service delivery) qualitative methods are used in ways designed to explore patient/carers' experiences in an open unrestricted manner—content being determined by what is important to them. Unlike data elicited from feedback types in Category 2, methods used within Category 3 are designed to elicit rich and collated *data sets ready to use*.

PE types within this category also come with varying levels of guidance for linking collection of feedback to QI techniques. Some advocate linking feedback directly into continuous learning process (action research within Patient Journeys^55^; metrics collected elsewhere are used to track progress within Kinda Magic^56^; mainstream QI approaches such as PDSA are used in 15 Steps and Always Events^58^, ^59^). EBCD/AEBCD^57^ recommends collecting qualitative feedback of impact to assess perceptions of how the service has changed and also suggests collecting other measures about the change, for example cost‐savings to a service. Three also stand out for the way they use feedback as data for problem definition. Within EBCD/AEBCD and Always Events, feedback is interpreted together with staff and patients/carers in a process of co‐design so that contextual meaning informed by those who work in the service can be added. Patient Stories^60^ offer less in the way of prescribed QI process than the others, focusing only on one aspect—the presentation of the story to people who can potentially make changes as a result (often Boards). Subsequent change techniques are implied but not defined.

Finally, we identify a fourth category of miscellaneous “Other” with two types of feedback^61^, ^62^: FFT^61^ and HowRWe^62^ do not fit any of the above three categories. They are both surveys that hospitals can initiate, asking standardized questions, but unlike those surveys in Category 1, they are not designed to capture large quantities of data (lots of questions) infrequently, but instead they are very short and designed to be used more frequently, potentially providing a more continuous flow of PE feedback. In both cases, their responses are requested on a scale (positive to negative), allowing qualitative comments to be added, and they can both be applied to any type of health‐care setting. This is where their similarities end however. The nature of the questioning is very different. FFT only has one question: *How likely are you to recommend our service to friends and family if they needed similar care or treatment? *This is noticeably different to HowRWe which uses its very short design (four questions) to ask specific things about kind treatment, listening, promptness and organization, more akin to the content of surveys in Category 1. The data arising from the HowRWe standardized questions are validated to provide comparable data over time and between areas, whereas the data arising from the FFT standardized question are not. Potentially then, the HowRWe tool has more obvious potential for measurement and monitoring of trends within QI overtime and between areas than FFT but it is FFT that is mandatory in England whereas HowRWe is a voluntary tool and therefore much less widespread. The qualitative comments arising from both of these tools can be likened to the data arising from the feedback types included in Category 2: qualitative and context‐specific therefore holding potential to be used to guide local‐level improvement, however, they are not provided as collated data ready to be used, and the steps to enable them to be used as data are not specified.

## 4 DISCUSSION

In this paper, we have responded to demands made for PE feedback to be used more effectively in QI^4^, ^5^, ^6^ by conducting a scoping review and characterization exercise of different PE feedback types, to highlight their various potential roles. This builds on recent attempts to distinguish between roles depending on the nature of data produced,^19^, ^20^, ^21^, ^22^, ^23^ which we believe can be helpfully linked to grounding concepts of measurement within QI.^24^ Our scoping review identified 37 different types of PE feedback “on offer” to staff within UK hospitals. Using a consensus exercise, we drafted a list of characteristics that we believed to be important indicators of potential roles for each type. Using these characteristics to assess each type, we arrived at four distinct categories that we named: “Hospital‐initiated quantitative surveys”; “Patient‐initiated qualitative feedback”; “Hospital‐initiated qualitative feedback”; and “Other.” We have described above the nature of each of these categories with reference to roles within QI. In addition, we make the following observations:

### 4.1 Mandated sources currently provide limited value within QI

Of the mandated PE feedback types available, none of these would appear immediately suitable for informing and monitoring local improvement process (eg, ward level). Mandated feedback currently comprises quantitative survey data (the national inpatient surveys for whole organizations,^26^, ^27^, ^28^, ^29^, ^30^, ^31^ A&E^28^, ^32^ and maternity departments^28^, ^33^), complaints and liaison service data^44^, ^45^, ^46^, ^47^, ^48^, ^49^, ^50^ one form of online feedback (NHS Choices^51^) and the Friends and Family Test results.^61^ The large quantitative surveys—many validated for representativeness with large samples—serve an accountability purpose but do not provide locally relevant data that are accessible to those who need it, in a timely manner^21^, ^63^ that would be required for informing and monitoring QI process. The FFT quantitative test^9^ appears to seek to address both requirements. Its qualitative, locally applicable information could, in principle, be used to inform what needs to improve but this proposal is fiercely questioned,^10^ described as a laudable ambition thwarted by an overemphasis on achieving acceptable response rates at the expense of considering and utilizing the qualitative comments effectively. The lack of standardization with respect to administration of its single quantitative question has also cast doubts on its suitability as a monitoring tool over time. As the surveys are not providing everything required for QI, there is increasing interest in the use of other mandated feedback—from complaints and liaison services—by coding and theming into data sets.^11^ However, there are challenges to these proposals,^12^ relating to system practicalities (collation of case‐by‐case complaints), the nature of the story told (complex and difficult to code) and availability (often infrequent and inconsistent in style). In short, seen from a QI perspective, mandatory PE data (national surveys, FFT, complaints/concerns and NHS Choices), currently appear to offer little ready‐to‐use data, with respect to informing and monitoring local PE improvement.

### 4.2 Other types of feedback offer potential

Other, non‐mandated types of feedback are available should hospitals wish to use them but an understanding of their potential uses is in relative infancy. Hospitals could use voluntary surveys of Category 1—these offer more granular data and can be used more flexibly if analytical capability exists.^23^ They could use the patient‐initiated qualitative types (eg, complaints, comments, social media reviews) which, due to their spontaneous nature, arguably tap into patients own concerns more readily than anything requested from the health‐care organizations themselves. Indeed, systems for harnessing such sources are emerging and include the development of dedicated websites for encouraging and organizing this feedback (eg, Care Opinion,^54^ iWantGreatCare,^52^ dedicated Facebook pages for some hospitals/wards). There is also an emerging interest in harnessing “the cloud of patient experience” from social media that could exist independently of any hospital systems just because the public use these platforms to discuss their hospital services (eg, Twitter, Facebook, Google).^64^ Finally, Category 3 (hospital‐initiated qualitative types) offer something else again—processes for not only collection of feedback but for the development of shared meanings about issues of importance, in a cooperative approach involving patients and staff, and in many cases, processes of action and reflection as a response to identified priorities.

### 4.3 Beyond the concept of PE metrics

In traditional QI theory,^24^ all three faces of measurement (accountability, improvement and universal knowledge) imply the need for data that contain objective metrics that can be tracked for changes over time. The nature of Category 3 provides some important insights about how PE feedback can be conceptualized within a QI process. Feedback collected through Category 3 methods elicits rich, open‐ended information, and staff are supported to engage meaningfully with these concerns as part of a continual learning process about how services can be improved as a result. In these approaches, feedback is not so much viewed as static metrics (objective data) but, linked to the concept of “soft intelligence”,^16^ it is viewed as a mechanism for disrupting staff assumptions and making space for patient/carer perspectives. For example, within two techniques (EBCD/AEBCD and Always Events), the aim is for staff and patients/carers to develop shared meanings from the feedback and within EBCD/AEBCD specifically; this is described as a co‐design and co‐creation process involving techniques that aid critical, collective reflection.^65^ Clearly, within this broader interpretation of feedback, the concept of triangulation introduced above^5^ as the basis through which to view multiple sources together is limited: some feedback provides metrics that may be identifiable in different sources, but some sources (eg, Category 3) will not elicit these metrics and are performing a function more akin to soft intelligence.^16^

### 4.4 Limitations

Due to the flexible approach taken to search terms, our scoping review may not have revealed all potential feedback types available in UK hospitals. Also, due to the sometimes subjective nature of these search terms, a repeat exercise by others may not yield exactly the same results. Our characterization exercise was based on best understanding at the time—at a different point in time, other characteristics could have been chosen. For example, we included a characteristic called “Supported by hospital system” to refer to whether or not the hospital invited, encouraged or organized this feedback. Since then, the term SSS (sanctioned, solicited, sought) has been introduced^14^ to distinguish between online feedback that organizations support, and that which exists independently, and such a term may have provided more clarity if we had been able to use it. When categorizing based on our characteristics, some subjective decisions were also made. In some cases, there was ambiguity and we used our characteristics list as a sensitizing framework rather than an absolute.

## 5 CONCLUSION

Our scoping review has confirmed that there are many different types of PE feedback available, or potentially available, within UK hospitals that appear to reflect a very worthwhile ambition to ensure patient/carer voice influences improvement of services. However, our characterization and categorization study has revealed that within these types, there are currently no “ready‐to‐use” data sets for informing and monitoring improvements to PE, apart from mandated data relating to high‐level organizational trends. Many decisions therefore have to be made about the extent to which hospitals engage with different types of PE feedback for their improvement initiatives. The categories we have introduced highlight the important differences to consider which could aid these decisions in hospitals and potentially in other health‐care environments. We know hospital teams are already struggling to handle feedback that they are mandated to collect^8^; therefore, informed decisions with respect to these options are crucial. To support this, we propose further analysis and conceptual development of the role of PE feedback within QI, and that the categories we present in this paper can be used to inform this process.

## CONFLICT OF INTEREST

All authors declare that they have no conflict of interest.

## AUTHORS’ CONTRIBUTIONS

CM oversaw study design and coordinated, conceptualized and wrote the paper with the support of RP, and will act as overall guarantor. RP and LH conducted the scoping review, coordinating input from Kathryn Melling. RL and LS convened and led the consensus team that developed the characteristics list and categories used to sort the findings of the scoping review. All authors contributed to the conceptualization and writing of the manuscript drafts, revised the manuscript critically for important intellectual content and gave final approval of the version to be published.

## ETHICAL APPROVAL

No ethical approvals were required to conduct this exercise.
