# Supplementary material for: Patient experience feedback in UK hospitals: What types are available and what are their potential roles in quality improvement (QI)?
Source: Health Expect. 2019 Apr 23;22(3):317–26. doi: 10.1111/hex.12885 (PMC6543142; doi:10.1111/hex.12885)
Supplement: Supplementary file 1 [file HEX-22-317-s001.docx]

The following changes have been made to the Appendices:

1) Table 1c has been renamed: *Hospital-initiated qualitative feedback*

2) 2 items have been removed from Table 1c: Emotional Touchpoints and Discovery interview

3) 1 item has been added to Table 1c: Patient Stories

### **Appendix 1: Types of patient experience feedback**

|  | **Data type** | **Level of applicability** | **Evidence for validity** | **Timing of feedback collection** | **Modes of feedback collection** | **Requirement** | **Supported by hosp system** | **Timeliness of feedback** | **Regularity of feedback** | **Who initiates feedback?** | **Who provides feedback?** | **Role in QI** |
| --- | --- | --- | --- | --- | --- | --- | --- | --- | --- | --- | --- | --- |
| NHS Adult Inpatient Survey | Quant (+ comments) | Hospital | Validated | Post-discharge | Survey (Paper) | Mandated | Yes | Delayed | Annual or bi-annual | Service Provider (at any level) | Patient | Data |
| NHS A&E survey | Quant (+ comments) | Service or specialty | Validated | Post-discharge | Survey (Paper) | Mandated | Yes | Delayed | Annual or bi-annual | Service Provider (at any level) | Patient | Data |
| NHS Maternity Services survey | Quant (+ comments) | Service or specialty | Validated | Post-discharge | Survey (Paper) | Mandated | Yes | Delayed | Annual or bi-annual | Service Provider (at any level) | Patient | Data |
| Scottish Inpatient Patient Experience Survey | Quant (+ comments) | Hospital | Validated | Post-discharge | Survey (Combination) | Mandated | Yes | Delayed | Annual or bi-annual | Service Provider (at any level) | Patient | Data |
| Scottish Maternity Care Survey | Quant (+ comments) | Service or specialty | Validated | Post-discharge | Survey (Combination) | Mandated | Yes | Delayed | Annual or bi-annual | Service Provider (at any level) | Patient | Data |
| Inpatient Patient Experience Survey 2014 (Northern Ireland) | Quant (+ comments) | Hospital | Validated | Post-discharge | Survey (Paper) | Mandated | Yes | Delayed | Ad hoc (run once in 2014) | Service Provider (at any level) | Patient | Data |
| Your NHS Wales Experience Questionnaire | Quant (+ comments) | Either | Validation ongoing | Either | Survey (Combination) | Not mandated (strongly recommended by NHS Wales) | Yes | Delayed | Not specified | Service Provider (at any level) | Patient | Data |
| Picker Patient Experience Questionnaire PPE-15 | Quant | Either | Validated | Post-discharge | Survey (Paper) | Voluntary | No | Delayed | Ad hoc | Service Provider (at any level) | Patient | Data |
| Patient Experience Questionnaire  (New models study) | Quant (+ comments) | Service or specialty | Not validated | Either | Survey (Paper) | Voluntary | No | Delayed | Ad hoc | Service Provider (at any level) | Patient | Data |
| Oxford Patient Involvement & Experience Scale (OxPIE) | Quant | Either | Validated | Either | Survey (Paper) | Voluntary | No | Delayed | Ad hoc | Service Provider (at any level) | Patient | Data |
| Newcastle Satisfaction with Nursing Scale | Quant | Either | Validated | Either | Survey (Paper) | Voluntary | No | Delayed | Ad hoc | Service Provider (at any level) | Patient | Data |
| Intensive Care Experience ICE Questionnaire | Quant | Service or specialty | Validated | Either | Survey (Presume paper) | Voluntary | No | Delayed | Ad hoc | Service Provider (at any level) | Patient | Data |
| Patient Evaluation of Emotional Care during Hospitalisation (PEECH) | Quant (+ comments) | Service or specialty | Validated | In situ | Survey (Paper) | Voluntary | No | Delayed | Ad hoc | Service Provider (at any level) | Patient | Data |
| Urgent Care System Questionnaire | Quant | Service or specialty | Validated | Post-discharge | Survey (Telephone) | Voluntary | No | Delayed | Ad hoc | Service Provider (at any level) | Patient | Data |
| Patient Career Diary | Quant (+ comments) | Either | Validated | Either | Survey (Paper) | Voluntary | No | Delayed | Ad hoc | Service Provider (at any level) | Patient | Data |
| VOICE survey | Quant (+ comments) | Either | Validated | In situ | Survey (Paper) | Voluntary | No | Delayed | Ad hoc | Service Provider (at any level) | Patient | Data |
| Hospital Care & Discharge: Patients & Carers Opinions | Quant (+ comments) | Hospital | Not validated | Post-discharge | Survey (Paper) | Voluntary | No | Delayed | Ad hoc | Service Provider (at any level) | Either | Data |

*Appendix 1a: Hospital-initiated quantitative surveys*

|  | **Data type** | **Level of applicability** | **Evidence for validity** | **Timing of feedback collection** | **Modes of feedback collection** | **Requirement** | **Supported by hosp system** | **Timeliness of feedback** | **Regularity of feedback** | **Who initiates feedback?** | **Who provides feedback?** | **Role in QI** |
| --- | --- | --- | --- | --- | --- | --- | --- | --- | --- | --- | --- | --- |
| Patient Advice and Liaison Services | Qual | Any | N/A | Either | Internal hospital forms (web/paper) | Mandatory | Yes | Real time | Ad hoc | Patients/carers | Either | Data |
| Feedback cards e.g. Points of You | Qual | Any | N/A | Either | Internal hospital forms (web/paper) | Voluntary | Yes | Real time | Ad hoc | Patients/carers | Either | Data |
| Formal complaints | Qual | Any | N/A | Either | Internal hospital forms (web/paper) | Mandatory | Yes | Real time | Ad hoc | Patients/carers | Either | Data |
| Informal feedback e.g. compliments | Qual | Any | N/A | Either | Internal hospital forms (web/paper)  *thankyou cards | Voluntary | No | Real time | Ad hoc | Patients/carers | Either | Data |
| NHS Choices | Qual  (+rating stars) | Any | N/A | Either | External (web) | Mandatory  formal support | Yes | Real time | Ad hoc | Patients/carers | Either | Data |
| Care Opinion (was Patient Opinion at time of search) | Qual | Any | N/A | Either | External (web) | Voluntary | Both | Real time | Ad hoc | Patients/carers | Either | Data |
| iWantGreatCare | Qual (+rating stars) | Any | N/A | Either | External (web) | Voluntary | No | Real time | Ad hoc | Patients/carers | Either | Data |
| Mumsnet | Qual | Any | N/A | Either | External (web) | Voluntary | No | Real time | Ad hoc | Patients/carers | Either | Data |
| Twitter | Qual | Any | N/A | Either | External (web) | Voluntary | No | Real time | Ad hoc | Patients/carers | Either | Data |
| Google reviews of hospitals | Quant (+ comments) | Any | N/A | Either | External (web) | Voluntary | No | Real time | Ad hoc | Patients/carers | Either | Data |
| Facebook set up by ward/hospital | Qual | Any | N/A | Either | External (web) | Voluntary | Yes | Real time | Ad hoc | Patients/carers | Either | Data |
| Facebook (general) | Qual | Any | N/A | Either | External (web) | Voluntary | No | Real time | Ad hoc | Patients/carers | Either | Data |

*Appendix 1b: Patient-initiated feedback processes (*General knowledge not returned in formal review)*

|  | **Data type** | **Level of applicability** | **Evidence for validity** | **Timing of feedback collection** | **Modes of feedback collection** | **Requirement** | **Supported by hosp system** | **Timeliness of feedback** | **Regularity of feedback** | **Who initiates feedback?** | **Who provides feedback?** | **Role in QI** |
| --- | --- | --- | --- | --- | --- | --- | --- | --- | --- | --- | --- | --- |
| Patient Stories | Qual | Any | N/A | Either | Qual research methods | Voluntary | Yes | Delayed | Ad hoc | Service provider | Either | Data |
| Kinda Magic approach | Mixed | Service or specialty | N/A | In situ | Qual research methods | Voluntary | Yes (Peninsula Trust) | Delayed | Missing | Service provider | Patient | Data +QI |
| Patient stories of care experience (EBCD & Accelerated EBCD) | Qual | Service or specialty | N/A | Either | Qual research methods | Voluntary | No | Delayed | Ad hoc | Service provider | Either | Data +QI |
| Patient journey (Action research Baron) | Qual | Service or specialty | N/A | Either | Qual research methods | Voluntary | No | Delayed | Ad hoc | Service provider | Patient | Data +QI |
| Always Events | Qual | Service or specialty | N/A | In situ | Qual research methods | Voluntary | *Promoted by NHSE | Delayed | Ad hoc | Service provider | Either | Data +QI |
| Fifteen Steps challenge | Qual | Service or specialty | N/A | In situ | Qual research methods | Voluntary | *Promoted by NHSE | Delayed | Ad hoc | Service provider | Observer* | Data +QI |

*Appendix 1c: Hospital-initiated qualitative feedback*

|  | **Data type** | **Level of applicability** | **Evidence for validity** | **Timing of feedback collection** | **Modes of feedback collection** | **Requirement** | **Supported by hosp system** | **Timeliness of feedback** | **Regularity of feedback** | **Who initiates feedback?** | **Who provides feedback?** | **Role in QI** |
| --- | --- | --- | --- | --- | --- | --- | --- | --- | --- | --- | --- | --- |
| HowRwe (How are we doing) | Quant (+ comments) | Service or specialty | Validation ongoing | In situ | Survey (Combination) | Voluntary | No | Real time | Continuous | Service Provider (at any level) | Patient | Data |
| Friends & Family Test | Quant (+ comments) | Either | Not validated | Either | Survey (Combination) | Mandatory | Yes | Real time | Continuous | Service Provider (at any level) | Patient | Data |

*Appendix 1d: Other*
